# Computational Modeling and Experimental Validation of Variabilities in Chemical Vapor Deposition of Graphene on Metals

**DOI:** 10.1002/smll.202513297

**Published:** 2026-05-11

**Authors:** Tanuj Joshi, R. K. Singh Raman, Yiannis Ventikos

**Affiliations:** ^1^ Department of Mechanical and Aerospace Engineering Monash University Melbourne Australia; ^2^ Research in Advanced Materials Alloys & Nanosystems (RAMAN) Lab Monash University Melbourne Australia

**Keywords:** chemical vapor deposition (CVD), computational fluid dynamics (CFD), graphene coating, raman spectroscopy, scanning electron microscopy (SEM), wall shear stress (WSS)

## Abstract

Achieving laterally uniform graphene coatings via low‐pressure chemical vapor deposition (LPCVD) remains challenging due to substrate‐scale variations in near‐wall transport that govern precursor renewal and local growth. Here, transient 3D CFD is coupled with spatially resolved characterization to examine how substrate inclination reorganizes near‐wall transport in a hot‐wall quartz‐tube LPCVD reactor and its influence on graphene thickness uniformity. Across four substrate tilt angles (9°, 21°, 33°, and 45°), uniform coatings emerge not from maximizing flow intensity, but from establishing a laterally distributed near‐surface transport field without significant downstream shielding. Shallow inclination (9°) produces weak surface‐parallel transport and thicker boundary layers. In contrast, steep inclination (45°) induces strong but highly localized acceleration followed by wake‐driven transport heterogeneity. Intermediate inclinations (21°–33°) yield more balanced near‐surface velocity and wall shear stress distributions, promoting spatially uniform precursor renewal across the substrate surface. Raman mapping and SEM‐based morphology analysis corroborate these transport trends, confirming reduced spatial segregation and improved thickness coherence within the 21°–33° inclination window. These findings establish a transport‐based framework for interpreting substrate orientation effects in LPCVD graphene growth and provide reactor‐level guidance for achieving uniform coatings in comparable hot‐wall LPCVD systems.

## Graphical abstract

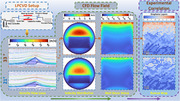

## 1 Introduction

Remarkable physical and chemical properties of graphene account for its emergence as the most investigated material of recent times. Owing to its unique combination of exceptional chemical stability, impermeability, and mechanical toughness [1]. Graphene is therefore a promising material for barrier coatings for metals and alloys against degradation in aggressive environments (corrosion) [2, 3, 4, 5]. However, graphene in such applications needs to be defect‐free and provide complete and continuous coverage of the metallic surface with a robust coating. *Chemical Vapor Deposition* (CVD) is among the few techniques that can develop pristine graphene over reasonably large areas, and hence, CVD may be attractive for industrial‐scale implementation of graphene coatings. Variations in the quality and robustness of CVD‐grown graphene reported across studies have led to considerable differences in the degree of corrosion resistance achieved. In this context, in one study, CVD graphene is reported to improve the corrosion resistance of copper by more than 2 orders of magnitude [1] to just an order of magnitude [6], whereas that improvement was reported to be insignificant in another study [7]. Such variabilities have comprehensively been attributed to the defective content and robustness of graphene in the corresponding studies. It may be fair to say that it is challenging to successfully develop a defect‐free and continuous *single layer* of graphene on large metallic surfaces of industry‐scale dimensions. However, it has been demonstrated that developing multilayer graphene (MLG) may circumvent the challenges of defects in single‐layer graphene, since the defects and discontinuities in the first layer could be masked by the subsequent layer(s). It has been mechanistically well‐established that Ni is more amenable to developing MLG by CVD [8], and hence, Ni and Ni‐based alloys have been pursued more frequently for graphene coatings for corrosion resistance [9, 10].

Although CVD has been widely pursued for graphene synthesis on metal substrates, the process remains highly sensitive to subtle variations in parameters such as flow of the precursor, temperature, gas concentration, and others [1]. Even minor fluctuations in these characteristics have been shown to cause nonuniform coverage, adlayer formation, and structural defects, thereby posing major challenges for reproducibility and large‐scale applications in areas such as flexible electronics and corrosion protection [11]. In this context, the orientation of a metallic substrate with respect to the precursor gas flow in a CVD reactor has been shown to considerably influence the flow characteristics of the incoming precursor gas stream and homogeneity of the precursor distribution over the metal surface, thereby influencing the defect density and mechanical robustness of the resulting graphene [8]. Such variations can profoundly influence local velocity, boundary‐layer thickness, wall shear stress (WSS), and temperature gradients, each of which will have a bearing on the effectiveness of CVD.

As also alluded to earlier, the flow dynamics in the CVD chamber are dictated not only by flow rate and gas pressure but also by geometric configuration and arrangements, including how the substrate is placed relative to the incoming gas stream. Substrate tilt modifies boundary‐layer thickness, WSS, and the local temperature field, which collectively determine the balance between carbon precursor delivery and hydrogen etching at the surface. Prior studies have demonstrated that reactor configuration and substrate tilt can directly influence thin‐film uniformity by shaping gas flow and heat distribution across the substrate [12]. Variations in placement and reactor geometry can further generate local gradients in precursor delivery, leading to spatial variations in graphene thickness [13]. In addition, optimized confinement and reactor geometries can regulate gas exchange and precursor distribution, thereby promoting more uniform large‐area graphene growth [14]. To rationalize these effects, Khanafer et al. [15] performed computational analyses of heat and mass transport in a converging CVD channel with a heated susceptor. It was demonstrated that substrate tilting suppresses recirculation, thins the boundary layer, and enhances mass transfer to the surface. At higher Reynolds numbers, optimized tilt angles of approximately 6°–9° eliminated vortical structures and increased precursor flux, resulting in more uniform films. This framework has played an important role in advancing transport‐informed CVD process design. Building on this concept, Zhang et al. [16] modeled graphene growth on Cu substrates and demonstrated that increasing the tilt to 30°–45° optimized boundary‐layer dynamics and exposed larger substrate areas to a homogeneous precursor gas supply, thereby promoting predominantly monolayer or few‐layer graphene. To improve large‐area coverage of high‐quality single‐layer graphene, Huet et al. [17] employed a closely packed vertical (90°) wafer configuration in a hot‐wall CVD reactor, which pronounced direct precursor impingement, thereby equalizing seeding density and growth rate across wafers. Underpinned by such prior findings, Arya et al. [8] performed low‐pressure CVD (LPCVD) on Ni substrates placed at 5° and 45° tilts of the substrate, and demonstrated that a 45° tilt enabled larger substrate areas to experience homogeneous supply of the precursor gas, and produced graphene coatings with considerably greater MLG compared to the substrate with 5° tilt. More recently, Khan et al. [18] combined computational fluid dynamics (CFD) simulations with atmospheric‐pressure CVD on Cu substrates at tilt angles of 8°, 15°, and 60°, and reported that an intermediate angle of 15° yielded high‐quality graphene with low defect density, supported by stable flow fields and thin as well as uniform boundary layers. In contrast, steeper tilts such as 60° induced flow separation, strong vorticity, and nonuniform boundary development, which led to defective and irregular graphene growth. Collectively, these studies highlight that moderate tilts stabilize boundary‐layer dynamics and promote uniform growth, while extreme orientations compromise coating quality due to adverse flow phenomena.

Despite the increasing use of computational fluid dynamics (CFD) to analyze flow behavior in LPCVD reactors, direct correlations between simulated transport fields and spatially resolved experimental measurements of coating uniformity remain limited. As a result, a gap persists between predicted hydrodynamic behavior and experimentally observed deposition outcomes. Bridging this gap is particularly important for graphene coatings used in corrosion protection, where even micrometer‐scale thickness variations or localized defects can accelerate degradation due to the cathodic nature of graphene. Although substrate inclination has been recognized as a factor influencing CVD growth behavior, its role has not been resolved within a framework that directly links inclination‐induced flow reorganization to experimentally observed coating uniformity. In this work, transient 3D CFD simulations are integrated with spatially resolved experimental characterization to examine how substrate inclination modifies near‐wall transport in a hot‐wall LPCVD reactor. Substrate tilt angles (9°, 21°, 33°, and 45°) are systematically investigated to quantify their influence on near‐surface velocity fields, wall shear stress distributions, and transport conditions at the growth interface. Complementary LPCVD experiments conducted under matched reactor conditions are analyzed using Raman mapping and SEM to quantify graphene thickness and morphological variations across the substrate. By directly correlating inclination‐induced transport reorganization with experimentally observed coating uniformity, this study establishes a mechanistically grounded and experimentally validated framework for understanding how substrate orientation governs graphene growth uniformity under LPCVD conditions. Collectively, the results move beyond treating substrate tilt as a purely empirical parameter and instead provide transport‐informed guidance for reactor‐substrate configurations in comparable hot‐wall LPCVD systems.

## 2 Materials and Methods

### 2.1 Substrate Preparation

Graphene films were synthesized on high‐purity nickel coupons (15 × 15 × 1 mm^3^; Puratronic 99.9945% metals basis, Alfa Aesar). The coupons were sequentially ground using silicon carbide abrasive papers of grit sizes P320, P800, P1200, and P2500 for 4 min each, followed by polishing with a 3 µm diamond suspension for 2 min to obtain a smooth surface finish. The polished samples were then ultrasonically cleaned in ethanol for 10 min, rinsed with deionized water, and dried under compressed dry air.

### 2.2 Graphene Growth Via LPCVD

Graphene growth was carried out in a LPCVD system equipped with a horizontal quartz‐tube furnace (inner diameter 23 mm; Lenton, U.K.). The Ni substrates were mounted on a ceramic holder inclined at a 9°–45° angle relative to the incoming gas stream. The quartz tube was evacuated to ∼10 mTorr using a dry scroll pump (nXDS10i, Edwards, Germany), after which a flow of Ar/H_2_ (85/15 vol%) at 250 sccm was introduced to establish a pressure of 1.18 Torr. The substrates were annealed at 1070°C for 40 min under this Ar/H_2_ atmosphere. Subsequently, n‐hexane vapor (1 sccm) was introduced as the carbon precursor, increasing the total pressure to 1.23 Torr. Graphene grew for 60 min under the described conditions, after which the furnace was cooled to below 100°C under Ar/H_2_ flow to prevent oxidation. The experimental parameters reported here follow emerging best‐practice reporting guidelines for CVD‐based 2D material synthesis [19].

### 2.3 Computational Modeling

Gas flow and heat transfer in the LPCVD reactor were modeled by solving the single‐phase, three‐dimensional laminar Navier–Stokes, continuity, and energy equations. Both steady‐state and transient simulations have been conducted.

Conservation of mass is enforced by the continuity equation:

(1) $$\frac{\partial \rho }{\partial t}+\nabla .\left(\rho u\right)=0$$

where: 𝜌 is density, and 𝑢 is the velocity vector.

Momentum conservation is captured by:

(2) $$\rho \left(\frac{\partial u}{\partial t}+\left(u.\nabla \right)u\right)=-\nabla p+\nabla .\left[\mu \left(\nabla u+{\left(\nabla u\right)}^{T}\right)\right]$$

where 𝑝 is the static pressure, and 𝜇 is the dynamic viscosity. This vector equation translates into three partial differential equations for the three spatial directions.

Thermal transport is described by:

(3) $$\rho c_{p}\left(\frac{\partial T}{\partial t}+u.\nabla T\right)=\nabla .\left(k\nabla T\right)$$

with 𝑇 temperature, 𝑐_𝑝_ specific heat, and 𝑘 thermal conductivity.

Equations of State supplemented by property models followed standard practice for LPCVD gases: ideal‐gas law for 𝜌(𝑇,𝑝); Sutherland's law for 𝜇(𝑇); and temperature‐dependent 𝑘(𝑇) and 𝑐_𝑝_(𝑇) [20]. The computational domain reproduced the quartz tube geometry (length 0.90 m, diameter 0.023 m) with a 0.30 m central heating zone, where Ni substrates were inclined at defined angles to assess orientation effects.

The flow was modeled under the present low‐Reynolds‐number regime (Re_D_ ≈ 8–10 based on tube diameter, and Re_L_ ≈ 3–5 including the characteristic substrate length), confirming laminar transport under the present LPCVD conditions. Temperature‐dependent density variation was explicitly resolved, with the gas density decreasing from 3.23 × 10^−3^ kg/m^3^ at the inlet (300 K) to 6.32 × 10^−4^ kg/m^3^ near the heated substrate region, corresponding to an approximately fivefold reduction, before partially recovering downstream as the gas cools. Continuum validity was assessed using the Knudsen number, $Kn=\frac{\lambda }{L}$, where the molecular mean free path is given by $\lambda =\frac{k_{B}T}{\sqrt{2}d^{2}P}$. For the present case, the mean fee path is λ = 1.8 × 10^−4^ m. Using the tube inner diameter as a characteristic length results Kn = 0.0078, confirming that the flow remains in the continuum regime at the reactor scale. Compressibility effects were assessed through the Mach number and remained below 0.2 throughout the domain; accordingly, a low‐Mach, variable‐density formulation was adopted to resolve the velocity and wall shear stress fields relevant to near‐wall transport. Simulations were performed using ANSYS Fluent 2024 R1 (ANSYS Inc., Canonsburg, PA, USA).

#### 2.3.1 Boundary Conditions

A continuous argon flow was introduced at the inlet with a volumetric flow rate of 250 sccm and an inlet temperature of 300 K. For computational simulations, the carrier gas was approximated as pure argon to represent the dominant transport properties of the experimental Ar/H_2_ mixture. The central heating zone of the CVD tube was maintained at a constant wall temperature of 1070°C (1343.15 K) to replicate deposition conditions. The reactor pressure was regulated at 165.5 Pa (≈1.23 Torr) to match the experimental LPCVD environment. Standard no‐slip, no‐flux boundary conditions were applied on all solid surfaces. A pressure‐outlet boundary condition was imposed at the downstream end of the computational domain to allow fully developed outflow while maintaining global pressure consistency.

#### 2.3.2 Meshed Model and Mesh Independence Test

The three‐dimensional computational domain representing the LPCVD setup was discretized using an unstructured tetrahedral mesh, with inflation layers introduced near solid boundaries to adequately resolve near‐wall gradients associated with boundary‐layer development [21, 22, 23, 24, 25]. Grid independence was assessed using four progressively refined meshes containing approximately 2.1, 3.3, 6.5, and 10 million elements (Figure S1a). Velocity profiles evaluated at diagnostic cross‐sections within the reactor showed negligible variation across mesh densities, confirming that the predicted flow field is insensitive to further grid refinement (Figure S1b,c). Based on this assessment, a mesh containing approximately 3.3 million elements was selected for subsequent simulations, providing a balance between numerical accuracy and computational efficiency. Temporal sensitivity analysis further confirmed that a time step of Δ*t* = 1 × 10^−6^ s is sufficient to resolve the dominant transient flow structures while maintaining stable convergence. Additional details of the mesh construction, grid‐convergence evaluation, and time‐step sensitivity analysis are provided in the Supporting Information.

#### 2.3.3 Transport‐Reaction Regime Analysis

To determine whether graphene growth under the present LPCVD conditions is governed primarily by precursor transport or surface reaction kinetics, an order‐of‐magnitude comparison between characteristic mass‐transport and reaction rates was performed. Under LPCVD graphene growth conditions (∼1000–1300 K), reported diffusion coefficients of hydrocarbon species in carrier gases are on the order of 10^−4^ m^2^.s^−1^, while characteristic surface decomposition rate constants are reported in the range of 0.02–0.05 m.s^−1^ depending on the precursor chemistry. Using a characteristic near‐wall transport length scale of approximately 1 mm extracted from the CFD boundary‐layer structure, the resulting Damköhler number $Da=\frac{k_{r}L}{D}$ is estimated to be ∼0.2–0.5 (see Section S2). Damköhler numbers smaller than unity indicate that the characteristic time scale of surface reactions is comparable to or longer than that of mass transport. Consequently, precursor delivery to the substrate surface can be significantly influenced by local transport processes rather than being controlled solely by reaction kinetics. Under these conditions, graphene growth in the present LPCVD system operates in a mixed transport‐reaction regime, where variations in the carrier‐gas flow field influence the spatial distribution of precursor flux at the substrate surface. The present CFD model, therefore, focuses on resolving the carrier‐gas flow field and the resulting near‐wall transport environment governing precursor renewal at the catalytic surface, while detailed reaction kinetics are treated as secondary to the transport variations induced by substrate inclination. Substrate inclination alters boundary‐layer development and near‐wall momentum transfer, thereby modifying the spatial distribution of precursor supply across the growth surface.

This transport‐mediated coupling between flow structure and precursor delivery provides the physical basis for analyzing the influence of substrate inclination on graphene growth uniformity in the following sections. It should be noted that the present CFD model is not intended to predict absolute graphene growth rates, but rather to resolve how substrate inclination reorganizes the carrier‐gas flow field and near‐wall transport environment that governs precursor renewal at the catalytic surface.

### 2.4 Characterization of Graphene

Raman spectroscopy was employed to verify the presence of graphene, and to determine the number of graphene layers (and to assess point defect density), using a Renishaw inVia Raman Microscope (Renishaw plc, Wotton‐under‐Edge, UK) equipped with a 514 nm (green) excitation laser (17.8 mW). Spectra were acquired in extended mode with a scan range of 100–4000 Raman shift (cm^−1^). The exposure time was 10 s with a single accumulation per spectrum. An Olympus 50× long working distance lens (8 mm focal length) was employed with a spot size of ∼3 µm. Raman spectra were acquired from 450 discrete points uniformly distributed across the 15 × 15 mm^2^ Ni substrate after CVD, and the collected data were subsequently used to construct spatial maps for evaluating the uniformity and quality of MLG growth.

Surface morphology and coating uniformity were examined using a Phenom XL benchtop scanning electron microscope (Thermo Fisher Scientific, USA) operated at 10 kV in secondary electron mode. SEM imaging was performed on graphene‐coated Ni substrates to evaluate surface topography and to assess coating continuity, including the presence of wrinkles, discontinuities, and pinhole‐like defects. Representative micrographs were acquired from multiple regions across each sample to capture spatial variability and enable qualitative comparison between substrate orientations.

## 3 Results and Discussion

### 3.1 Tilt‐Induced Reorganization of Near‐Wall Transport Conditions

**FIGURE 1:**
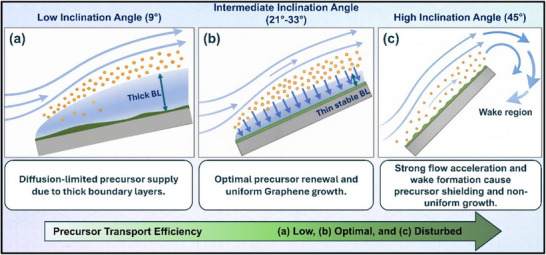
Schematic illustration of inclination‐controlled transport during LPCVD graphene growth. (a) At low inclination (9°), a thick boundary layer and diffusion‐limited precursor supply. (b) Intermediate inclination (21°–33°) generates a thinner and more stable boundary layer with distributed surface‐parallel flow, enabling efficient precursor renewal and uniform graphene growth. (c) At high inclination (45°), strong flow acceleration and wake formation produce precursor shielding and heterogeneous growth conditions.

All CFD simulations were performed under transient conditions to resolve the unsteady transport environment within the LPCVD reactor. The resulting velocity and wall shear stress fields were used to examine how substrate inclination redistributes near‐wall momentum transfer across the growth surface, thereby influencing precursor access, local residence conditions, and the spatial uniformity of graphene deposition. Figure 1 summarizes the transport mechanism by which substrate inclination governs graphene growth uniformity during LPCVD. At low inclination (9°) (Figure 1a), the flow remains weakly deflected over the substrate, producing a relatively thick boundary layer and limited precursor renewal near the growth surface. This diffusion‐dominated environment promotes localized variations in graphene thickness. At intermediate inclinations (21°–33°), the carrier gas accelerates smoothly along the exposed surface, generating a thinner and more stable boundary layer with laterally distributed surface‐parallel flow (Figure 1b). These conditions provide the most balanced precursor renewal and, therefore, the most uniform graphene growth environment. In contrast, at high inclination (45°), strong leading‐edge acceleration is followed by wake formation downstream, creating precursor shielding and spatially heterogeneous transport conditions that favor non‐uniform graphene thickness and morphology (Figure 1c).

#### 3.1.1 Effect of Substrate Tilt on Near‐Surface Transport

**FIGURE 2:**
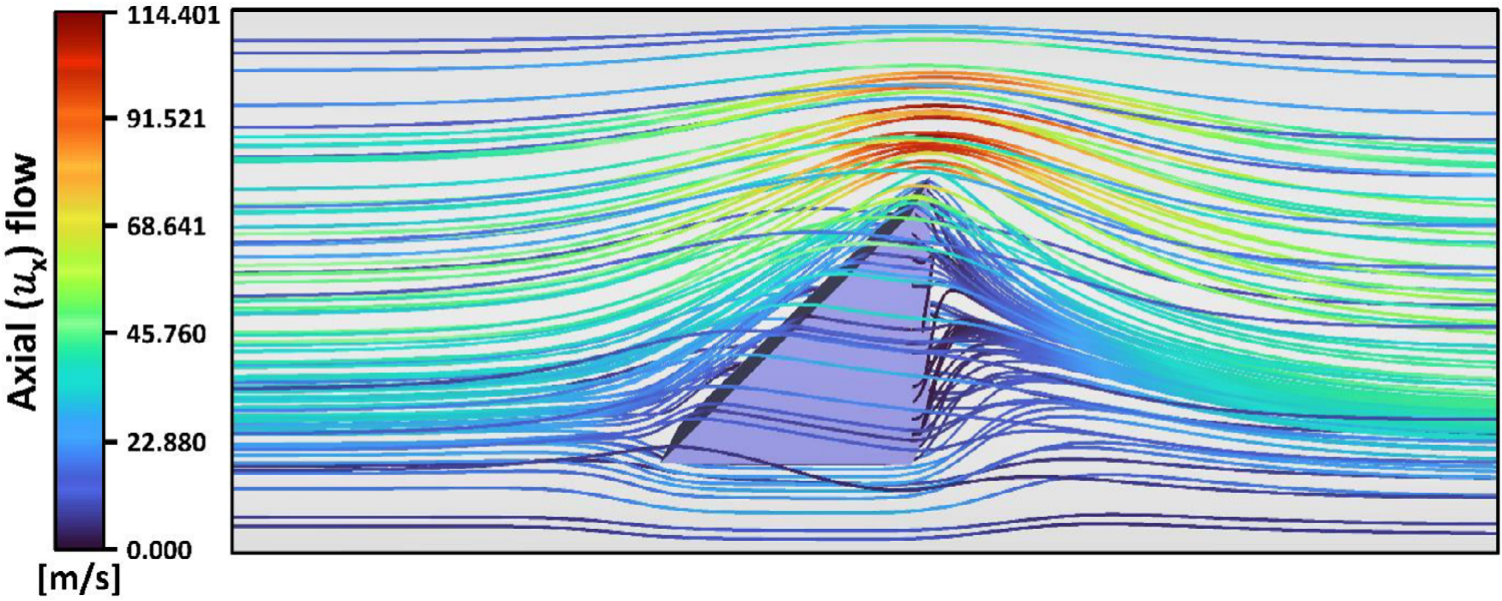
Representative carrier‐gas flow field over a 45°‐inclined Ni substrate during LPCVD, illustrating strong acceleration over the exposed leading surface and the formation of a downstream wake region. The resulting contrast between high‐renewal and shielded near‐wall transport zones highlights the mechanism responsible for spatial non‐uniformity in graphene growth.

Figure 2 illustrates the representative carrier‐gas flow field for the 45°‐inclined Ni substrate and highlights the near‐surface transport conditions governing spatial growth variations during LPCVD. Under the present low‐pressure environment (165.5 Pa), the incoming carrier gas enters with an average axial velocity of ∼7 m/s. As the flow encounters the inclined leading edge, it accelerates along the exposed surface and reaches a local maximum near the apex (∼114 m/s). This favorable‐pressure‐gradient‐driven acceleration results in streamline convergence, boundary‐layer thinning, and locally elevated wall shear stress, collectively promoting rapid gas renewal and enhanced convective transport near the exposed region of the substrate [26].

In contrast, the region beneath and downstream of the inclined substrate experiences pronounced flow deceleration and separation, leading to the formation of a low‐velocity wake characterized by recirculatory motion. The coexistence of these accelerated and wake‐dominated zones generates strong spatial variations in near‐wall transport conditions across the substrate surface. Regions exposed to rapid surface‐parallel flow experience shorter local residence times and more effective gas refreshment, whereas shielded wake regions undergo reduced convective renewal and modified precursor supply conditions. Consequently, the 45° configuration does not create a uniformly enhanced growth environment but instead partitions the substrate into high and low‐renewal domains. This transport heterogeneity provides a mechanistic basis for the experimentally observed spatial variations in graphene thickness and morphology across the inclined substrate.

**FIGURE 3:**
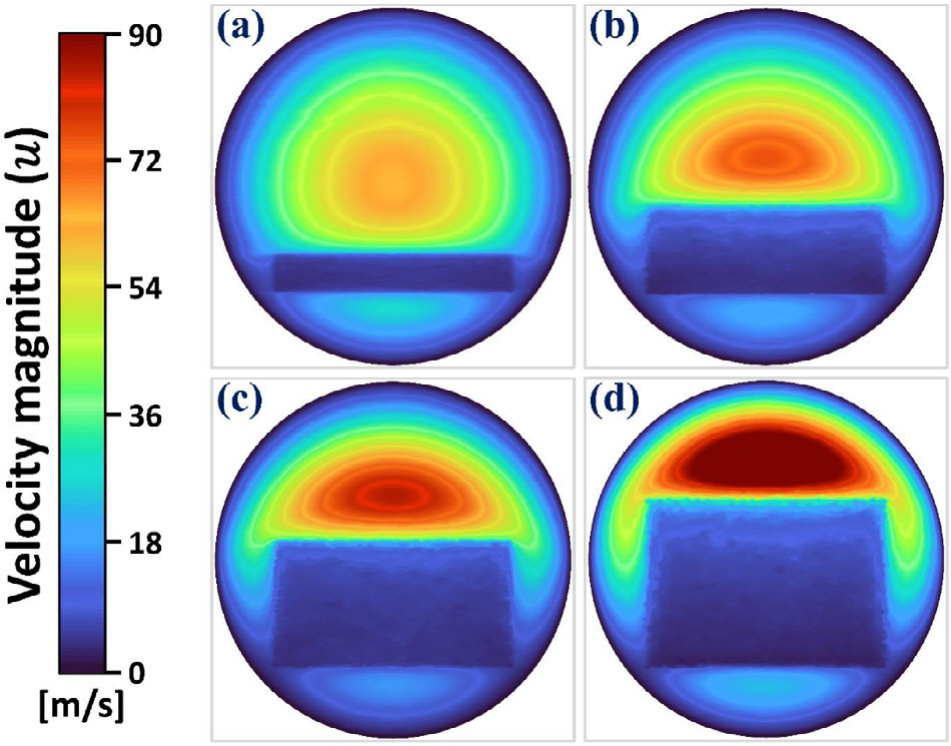
Instantaneous velocity magnitude contours extracted 0.5 mm above the inclined substrate from transient simulations for tilt angles of: (a) 9°, (b) 21°, (c) 33°, and (d) 45°. Intermediate tilt angles generate laterally distributed near‐surface acceleration across the exposed growth region, whereas shallow and steep inclinations produce weaker boundary‐layer transport and localized high‐velocity zones with downstream shielding, respectively.

Transient simulations were performed to evaluate how substrate inclination modifies the near‐surface velocity field governing precursor transport during graphene growth. Figure 3a–d presents instantaneous velocity magnitude contours extracted on a plane located 0.5 mm above the inclined substrate for tilt angles of 9°, 21°, 33°, and 45°, respectively. At a shallow inclination of 9° (Figure 3a), the incoming carrier gas undergoes only weak streamline deflection along the substrate surface, producing a relatively uniform but low velocity field across the growth region. Such conditions correspond to a thicker boundary layer and comparatively weak near‐surface convective transport and gas renewal, resulting in slower gas renewal near the substrate and increased local residence time. Increasing the tilt to 21° (Figure 3b) strengthens streamline compression over the exposed face and elevates surface‐parallel velocities while maintaining lateral continuity across the substrate‐facing region. The accelerated flow extends over a larger fraction of the exposed surface, indicating that a broader area experiences sustained convective renewal and more uniform precursor delivery. For the 33° tilt (Figure 3c), near‐surface velocities increase further and form an extended high‐velocity band over the upstream and central portions of the exposed region. Importantly, this acceleration remains spatially distributed rather than localized, suggesting efficient gas renewal over a comparatively large growth area. In contrast, the steep 45° configuration (Figure 3d) produces the strongest local acceleration but confines it to a compact high‐velocity zone near the leading edge, accompanied by sharp velocity gradients and downstream shielding. Consequently, despite the highest peak velocities, the resulting transport field becomes highly heterogeneous across the substrate surface, potentially leading to uneven precursor delivery and spatial non‐uniformity in graphene growth. Detailed downstream analysis of the velocity field, encompassing wake structures, vortex pairs, and flow separation, is presented in Figure S2a–d.

#### 3.1.2 Wall Shear Stress and Transport Uniformity

**FIGURE 4:**
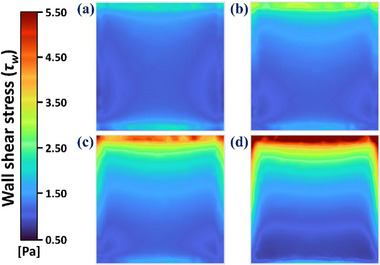
Wall shear stress (WSS) distributions over the inclined Ni substrate surface for tilt angles of (a) 9°, (b) 21°, (c) 33°, and (d) 45° in a hot‐wall LPCVD reactor. Increasing substrate inclination amplifies upstream shear and redistributes near‐surface stress, with moderate tilts producing broader shear zones and steep tilts generating localized shear ridges. These variations influence transport uniformity and precursor delivery at the growth interface.

Figure 4a–d presents the spatial distribution of wall shear stress (WSS), i.e., τ_*w*_ = μ[∇*u* + (∇*u*)^*T*^] · *n* − μ[*n* · {∇*u* + (∇*u*)^*T*^} · *n*]*n* distribution over the inclined Ni substrate for tilt angles of 9°, 21°, 33°, and 45°, respectively, providing the direct measure of near‐wall shear imposed by the carrier‐gas flow. Across all orientations, τ_*w*_ it is amplified at the upstream/leading‐edge region along with the lateral edges, consistent with streamline compression and edge‐driven shear amplification, while the central and downstream regions exhibit comparatively lower τ_*w*_. At the shallow inclination of 9° (Figure 4a), the WSS field is dominated by a broad low‐shear interior (∼1 Pa), with only a narrow upstream ridge of moderately elevated τ_*w*_ along the upstream edge. The lateral edges show weak‐to‐moderate intensification (∼2.5 Pa), while the central region exhibits a broad low‐shear plateau. Increasing the tilt to 21° (Figure 4b) strengthens the leading‐edge shear ridge and broadens its footprint across the upper portion of the substrate, producing a more continuous high‐ τ_*w*_ band (∼3 Pa) while maintaining relatively smooth lateral gradients over the central region. For the 33° inclination (Figure 4c), the upstream WSS amplification becomes more pronounced, with a continuous high τ_*w*_ band (∼4.5 Pa) extending across the leading edge and intensified corner regions at the lateral boundaries. Compared with 21° (Figure 4b), the transition from the high‐shear upstream band to the lower‐shear interior occurs over a larger portion of the surface, yielding a more structured τ_*w*_ gradient from the leading edge toward the downstream region. In the 45° configuration (Figure 4d), the leading‐edge WSS reaches its highest intensity and forms a compact, high‐contrast ridge (∼5.5 Pa) spanning the upstream boundary, with strong shear concentrations also apparent along the lateral edges and near the upper corners. Immediately downstream, τ_*w*_ decays sharply, producing the strongest spatial non‐uniformity among the four orientations. The spatial distribution of τ_*w*_ mirrors the near‐surface velocity organization in Figure 4, reflecting the dependence of wall shear on near‐wall velocity gradients: regions of accelerated surface‐parallel flow exhibit elevated τ_*w*_, whereas low‐velocity or shielded regions exhibit reduced shear.

Together, the velocity and WSS fields demonstrate that substrate inclination reorganizes the near‐wall transport environment in the LPCVD reactor, thereby governing precursor renewal and near‐wall mass transport across the growth surface. Intermediate tilt angles (21°–33°) generate laterally distributed surface‐parallel flow and more spatially uniform WSS across the growth surface, whereas shallow (9°) and steep (45°) inclinations produce weaker boundary‐layer transport or strongly localized high‐shear regions. These transport variations directly influence precursor renewal at the substrate surface and thereby control the spatial uniformity of graphene growth. Consistent with this mechanism, the experimentally observed Raman mapping for thickness distributions and SEM morphologies presented in Section 3.2 show the most uniform multilayer graphene formation at intermediate inclination angles, confirming the strong coupling between inclination‐driven flow organization and graphene coating uniformity.

### 3.2 Experimental Validation of Transport‐Mediated Graphene Growth Uniformity

Experimental measurements were performed to validate the transport‐driven trends predicted by the CFD simulations. Spatially resolved Raman spectroscopy and scanning electron microscopy (SEM) were employed to quantify graphene thickness uniformity and surface morphology as functions of substrate inclination. These measurements provide direct experimental evidence linking inclination‐induced modifications of the near‐wall flow field to the spatial organization of graphene growth across the substrate.

#### 3.2.1 Spectroscopic Evidence of Inclination‐Controlled Multilayer Graphene Formation

**FIGURE 5:**
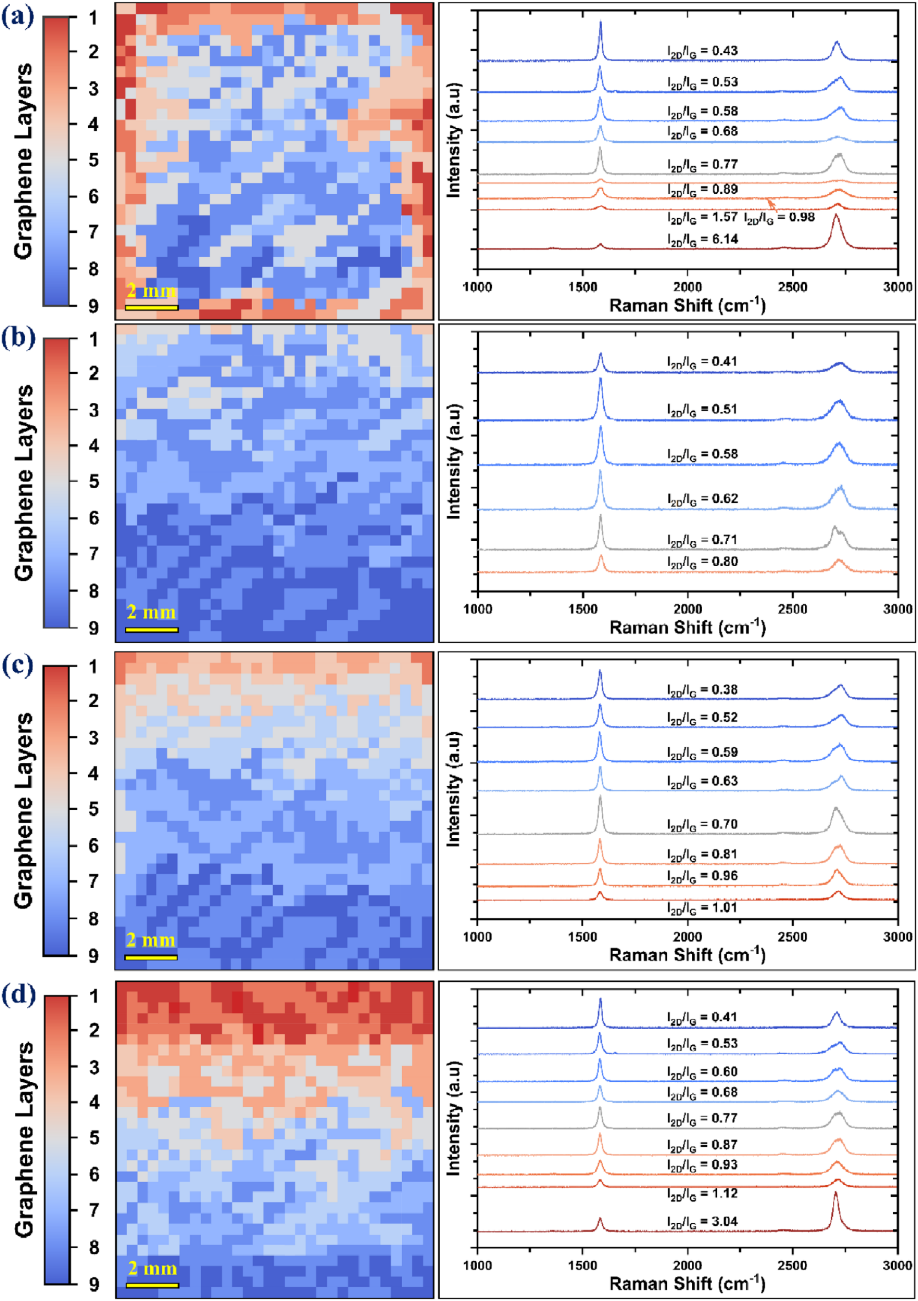
Spatial Raman maps (I_2D_/I_G_) indicating graphene layer distribution across a 15 × 15 mm^2^ substrate (left), and corresponding Raman spectra showcasing I_2D_ and I_G_ peaks with their ratio across the substrate (right) for (a) 9°, (b) 21°, (c) 33°, and (d) 45°.

Raman spectroscopy was employed to evaluate graphene coating uniformity and to determine whether the experimentally observed thickness distributions follow the inclination‐dependent near‐wall transport environments identified in Section 3.1. Spatial Raman maps acquired over a 15 × 15 mm^2^ area were generated from the I_2D_/I_G_ intensity ratio, while the accompanying spectra captured the corresponding variation of the 2D and G bands across selected surface locations [27]. These measurements provide a direct experimental basis for comparing graphene thickness distributions with the inclination‐induced differences in near‐surface velocity patterns and wall shear stress (WSS) revealed by the CFD analysis. For the shallow 9° inclination (Figure 5a), the Raman map exhibits pronounced spatial fluctuations in graphene thickness across the substrate. The representative spectra span a broad I_2D_/I_G_ range of 0.43–1.57, indicating substantial variability in multilayer graphene thickness across the mapped surface. In addition, an isolated domain with a markedly high I_2D_/I_G_ value of 6.14 indicates the presence of a mono‐layer graphene region within the broader multilayer background. This strong spatial heterogeneity is consistent with the flow dynamic environment established for the shallow‐inclination case. As discussed in Section 3.1.1, the 9° configuration produces weak surface‐parallel acceleration and a comparatively thick near‐wall boundary layer, while the associated WSS field remains low over most of the substrate interior (Figure 4a). Under such conditions, precursor renewal over the growth surface is less spatially organized, allowing local thickness variations to persist without strong upstream‐downstream homogenization. Increasing inclination to 21° (Figure 5b), both the Raman map and the representative spectra indicate markedly reduced variability relative to the 9° case. The I_2D_/I_G_ values cluster within a narrower range of 0.41–0.80, corresponding to approximately 5–9 graphene layers, and the spatial map reflects comparatively uniform multilayer coverage across the surface. This behavior is consistent with the transport environment identified in the CFD analysis, where the exposed substrate face experiences strong but laterally distributed surface‐parallel acceleration together with a broadened leading‐edge WSS band of moderate intensity. Such a flow field promotes more uniform near‐wall momentum transfer and boundary‐layer renewal across the growth surface, thereby suppressing isolated thickness extremes.

A similar trend is observed for the 33° inclination (Figure 5c), although with slightly broader variability than at 21°. The representative spectra span an I_2D_/I_G_ range of 0.38–1.01, corresponding to approximately 2–9 layers. However, unlike the 9° configuration, the 33° sample does not show isolated extreme thin‐domain signatures. Instead, thinner regions are preferentially localized toward the upstream portion of the substrate, where acceleration remains strongest, while downstream regions retain comparatively stable transport conditions. This behavior is consistent with the extended high‐velocity band and laterally distributed leading‐edge shear identified in Sections 3.1.1 and 3.1.2, which maintain much of the substrate within a relatively narrow WSS range (τ_w_ = 0.7–1.7 Pa). In contrast, the higher inclination 45° configuration (Figure 5d) exhibits the most pronounced thickness variability. The Raman spectra vary over an I_2D_/I_G_ range of 0.41–1.12, showing extended thinner regions adjacent to substantially thicker multilayer domains. An isolated domain with *I*_2D_/*I*_G_ = 3.04 further indicates the presence of a very thin monolayer graphene region. This behavior is fully consistent with the highly heterogeneous transport environment identified for the steep‐inclination case, where strong localized acceleration near the apex is followed by rapid downstream decay and extended low‐shear wake regions. Taken together, the Raman results confirm that intermediate inclinations (21°–33°) generate the most spatially balanced growth environment, whereas shallow and steep inclinations produce the largest thickness non‐uniformities through weak renewal and localized shielding, respectively.

#### 3.2.2 Morphological Evidence of Inclination‐Controlled Graphene Growth

**FIGURE 6:**
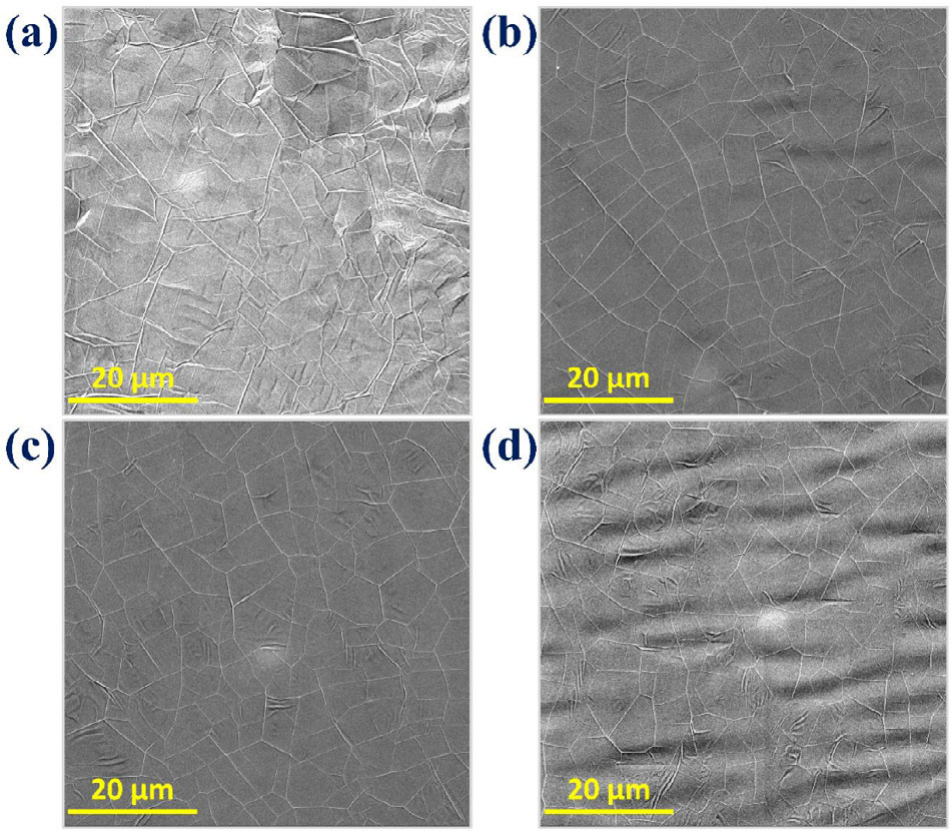
SEM micrographs of graphene coatings grown on Ni substrates at different substrate inclination angles: (a) 9°, (b) 21°, (c) 33°, and (d) 45°, illustrating inclination‐dependent variations in wrinkle network, fold density, and mesoscale corrugation of the graphene coating.

SEM micrographs provide complementary evidence of inclination‐dependent growth environments by revealing systematic variations in graphene surface morphology with substrate tilt (Figure 6a–d). Across all samples, the organization of wrinkle networks, fold density, and mesoscale corrugation features changes markedly with inclination, indicating that the local transport conditions during growth influence not only graphene thickness distribution but also the mesoscale structure of the coating. A shallow 9° tilt (Figure 6a) resulted in a highly dense population of intersecting wrinkles and short fold segments, forming an irregular network with pronounced spatial variability. The non‐uniform wrinkle spacing and frequent local fold clustering are consistent with a weakly renewed near‐wall environment, in which limited surface‐parallel transport permits localized growth heterogeneity to develop. The 21° configuration (Figure 6b) exhibits a markedly highly ordered morphology, with a continuous polygonal wrinkle network, more uniform spacing, and fewer localized folds. This indicates improved lateral continuity of the graphene coating, consistent with the more spatially balanced transport field identified in Sections 3.1.1 and 3.1.2. A similarly coherent morphology is observed at 33° tilt (Figure 6c), where wrinkle networks remain continuous over large areas with only modest variation in orientation and spacing. Localized folds are less frequent and do not organize into extended bands, confirming that near‐wall transport remains broadly distributed across the substrate. In contrast, the 45° tilt (Figure 6d) exhibits pronounced mesoscale corrugations and ridge‐like fold bands superimposed on the finer wrinkle network. These aligned, wave‐like features reflect strongly localized growth conditions, consistent with the CFD‐predicted combination of sharp near‐surface acceleration and downstream shielding.

**FIGURE 7:**
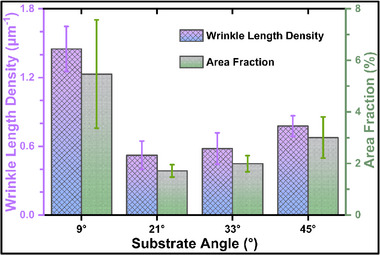
Quantitative variation of wrinkle length density (left axis) and wrinkled area fraction (right axis) for graphene coatings grown on Ni substrates at different inclination angles (9°, 21°, 33°, and 45°). Error bars denote the standard deviation from multiple SEM regions.

The quantitative evolution in wrinkle length density and wrinkled area fraction (Figure 7) is consistent with the surface morphologies observed in the SEM micrographs (Figure 6), confirming that substrate inclination systematically governs the development of wrinkle networks in the graphene coating. Such fragmented morphology reflects weak near‐wall transport conditions predicted by CFD at low inclination, where limited boundary‐layer renewal promotes localized growth variations also evident in the Raman thickness maps (Figure 5a). At 21° tilt, both wrinkle density and area fraction decrease substantially, accompanied by the emergence of longer and more continuous polygonal wrinkle networks with fewer localized folds (Figure 7). The improved spatial regularity indicates enhanced lateral continuity, consistent with the more uniform near‐wall transport. For the 33° tilt, both parameters increase moderately relative to 21° tilt, yet remain considerably lower than at 9° and 45°, indicating that the wrinkle network remains broadly distributed without strong localization. In contrast, the 45° sample exhibits elevated wrinkle density and area fraction together with greater scatter, consistent with the mesoscale corrugations and aligned fold bands visible in SEM.

### 3.3 Applicability and Limitations of the Geometry‐Based Design Principle

The experiments and simulations were conducted in a hot‐wall quartz‐tube LPCVD reactor operating under laminar, low‐Mach, variable‐density flow conditions with a confined substrate holder geometry. Under these conditions, substrate inclination primarily modifies near‐wall momentum transfer and boundary‐layer development, which together control precursor renewal and gas‐surface interaction across the growth surface. The optimal inclination window identified here (21°–33°) reflects the balance between surface‐parallel flow acceleration and downstream wake shielding established in the reactor geometry. In LPCVD systems with different tube diameters, confinement ratios (ratio of substrate holder length to reactor tube diameter), substrate arrangements, the flow field topology, and boundary‐layer recovery may change, potentially shifting the inclination at which near‐wall transport becomes most spatially uniform. In addition, the precursor gas composition employed in this work was optimized for the present reactor configuration to ensure stable graphene growth under these LPCVD conditions [8]. Variations in gas composition or operating regime can alter precursor decomposition, surface kinetics, and transport characteristics, and may therefore influence the growth response to substrate inclination. Accordingly, the present findings should be viewed as transport‐informed guidance for comparable hot‐wall LPCVD systems, and the inclination window identified here should be interpreted as a reactor‐specific transport optimum rather than a universal geometric rule for all CVD growth configurations.

The catalytic substrate also influences the extent to which inclination‐driven transport variations translate into observable growth non‐uniformity. Ni was selected in the present study because of its relatively high carbon solubility and dissolution‐segregation behavior, which enable multilayer graphene formation (1–9 layers) over a measurable thickness range, allowing spatial transport effects to be resolved with high sensitivity. In this context, Ni provides a suitable model system for establishing the relationship between near‐wall transport and coating uniformity. At the same time, the geometric mechanism, i.e., the redistribution of near‐surface momentum transfer and precursor renewal caused by substrate inclination, is not inherently specific to Ni. Prior works have also reported that geometric parameters in Ni‐based CVD systems [8] remain relevant for graphene growth on Cu and Cu–Ni alloy catalysts [28], indicating that reactor configuration and substrate placement can similarly influence gas‐flow distribution across different catalytic substrates. However, because catalytic activity, carbon solubility, and surface‐reaction pathways differ among catalyst systems, the observable impact on graphene thickness uniformity may vary depending on whether growth remains transport‐sensitive or becomes more strongly governed by surface kinetics. To isolate the role of transport‐driven effects in the present system, finely polished flat Ni coupons were employed, which is particularly important for protective graphene coatings, as residual oxides or irregular surface features can disrupt graphene continuity, weaken coating robustness, and promote localized degradation in aggressive electrolytes [7]. In real‐life applications, metallic substrates often exhibit surface roughness, texture, or three‐dimensional features that can locally perturb boundary‐layer development and nucleation behavior. Although the exact inclination response may therefore vary under non‐ideal conditions, the macroscopic transport mechanism identified here provides a useful framework for guiding reactor configuration and substrate orientation in practical growth environments. In systems where graphene growth becomes strongly reaction‐limited (e.g., at lower temperatures or with catalysts exhibiting slower precursor decomposition kinetics at the catalytic surface), the sensitivity of graphene thickness uniformity to hydrodynamic transport variations, and therefore to substrate inclination, could be correspondingly reduced.

## 4 Conclusions

This work establishes a mechanistically grounded understanding of how substrate inclination reorganizes near‐wall transport in a hot‐wall LPCVD reactor and governs the spatial uniformity of graphene coatings on Ni substrates under the present growth conditions. By integrating transient three‐dimensional CFD with spatially resolved Raman mapping and SEM characterization, the study directly correlates inclination‐dependent flow organization with precursor renewal at the growth interface and the resulting thickness and morphological uniformity. Shallow inclination (9°) produces weak surface‐parallel transport and thicker boundary layers, whereas steep inclination (45°) induces strong but highly localized acceleration followed by downstream shielding, leading to pronounced transport heterogeneity. In contrast, intermediate inclinations (21°–33°) generate more laterally distributed near‐surface velocity fields and balanced wall shear stress, promoting spatially uniform precursor renewal across the substrate. Consistent with these transport characteristics, Raman and SEM analyses confirm that intermediate inclinations yield the most continuous and morphologically uniform multilayer graphene coatings. These findings establish a direct link between inclination‐induced flow reorganization and experimentally resolved coating uniformity, providing a transport‐based framework for interpreting substrate orientation effects in LPCVD graphene growth. The resulting insights offer reactor‐level guidance for optimizing graphene coating uniformity in comparable hot‐wall LPCVD systems.

## Conflicts of Interest

The authors declare no conflicts of interest.

## Supporting information




**Supporting File**: smll73415‐sup‐0001‐SuppMat.docx.

## Data Availability

The data that support the findings of this study are available from the corresponding author upon reasonable request.
