# Supplementary material for: Computational Modeling and Experimental Validation of Variabilities in Chemical Vapor Deposition of Graphene on Metals
Source: Small. 2026 May 11;22(32):e13297. doi: 10.1002/smll.202513297 (PMC13244288; doi:10.1002/smll.202513297)
Supplement: Supplementary file 1 — Supporting File: smll73415‐sup‐0001‐SuppMat.docx. [file SMLL-22-e13297-s001.docx]

**Supplementary Information**

**Computational Modeling and Experimental Validation of Variabilities in Chemical Vapor Deposition of Graphene on Metals**Tanuj Joshi^1,2^, R.K. Singh Raman^1^, and Yiannis Ventikos^1,^*

^1^Department of Mechanical and Aerospace Engineering, Monash University, Clayton 3800, Australia

^2^Research in Advanced Materials, Alloys & Nanosystems (RAMAN) Lab, Monash University, Clayton 3800, Australia

**Section:S1 Computational Modeling**

We utilize the Control Volume methodology to discretize the above set of differential equations [1]. Pursuing pressure correction formalism, the PISO method (Pressure-Implicit with Splitting of Operators) was employed to ensure strong pressure-velocity coupling in transient laminar flows with steep thermal gradients. This algorithm uses predictor-corrector steps to enforce mass conservation and minimize pressure-velocity decoupling [2]. Gradients were evaluated using the least-squares cell-based method, which is robust for unstructured meshes and ensures accurate resolution of near-wall diffusion fluxes. Pressure was discretized with a second-order scheme to minimize truncation errors and generate smooth, physically realistic gradients, critical for accurately predicting velocity fields under low-pressure conditions. For momentum, central differencing was adopted to achieve second-order accuracy with minimum artificial diffusion, making it well-suited for laminar flows [1]. In contrast, convective energy transport was discretized using a second-order upwind scheme, which stabilizes scalar transport in the presence of steep thermal gradients while maintaining second-order accuracy. For the discretization of temporal derivatives in transient simulations, a second-order Crank-Nicolson implicit scheme was employed to ensure numerical stability and second-order accuracy in time integration [3].

The three-dimensional meshed model of the quartz tube reactor is shown in **Fig. S1a**. For discretization, 10-node tetrahedral elements were employed due to their suitability for capturing complex geometries and sharp edges. The final mesh achieved a minimum orthogonal quality of 0.87 to strengthen numerical stability and reliable CFD predictions. Inflation brick layers were introduced near the walls to resolve boundary-layer gradients more effectively, thereby improving the fidelity of the simulation results.

Multiple prior CFD studies [4–8] pursued mesh independence tests to ensure numerical accuracy without incurring prohibitive computational cost. To verify grid convergence in the present LPCVD model, four unstructured meshes comprising 2.1, 3.3, 6.5, and 10 million elements were evaluated, and mean axial velocity fields were interrogated at multiple diagnostic locations within the domain. Grid convergence was assessed at a cross-section located immediately upstream of the heated zone, where the flow is fully developed, see **Fig. S1b**. It was observed that across all mesh densities, the resulting velocity profiles were essentially indistinguishable and closely followed the analytical Hagen-Poiseuille solution for laminar pipe flow, with the expected ratio of maximum centerline velocity to mean inlet velocity of approximately two.

Similarly, **Fig. S1c** illustrates the resolution validation for the axial velocity (**𝑢*_x_***) obtained using the same mesh set (2.1-10 million elements), evaluated at a plane located ½D downstream of the substrate (D: quartz-tube diameter). The axial-velocity distribution is nearly indistinguishable across all meshes, confirming mesh independence and sufficient spatial discretization to accurately resolve near-wall gradients without numerical diffusion. The persistence of this profile collapse through the inflection region, where the flow undergoes substrate-induced acceleration and decay, indicates that all meshes consistently capture both boundary-layer shear and weak wake modulation. Temporal sensitivity analysis further showed that a time step of Δt = 1 × 10^-6^ s is sufficient to resolve the dominant unsteady flow structures while maintaining numerical stability, with all residuals converging below 10^-6^. Accordingly, the 3.3 million-element mesh and Δt = 1 × 10^-6^ s were selected for subsequent transient simulations, providing an optimal balance between computational efficiency and high-fidelity resolution of flow dynamics in the LPCVD system.


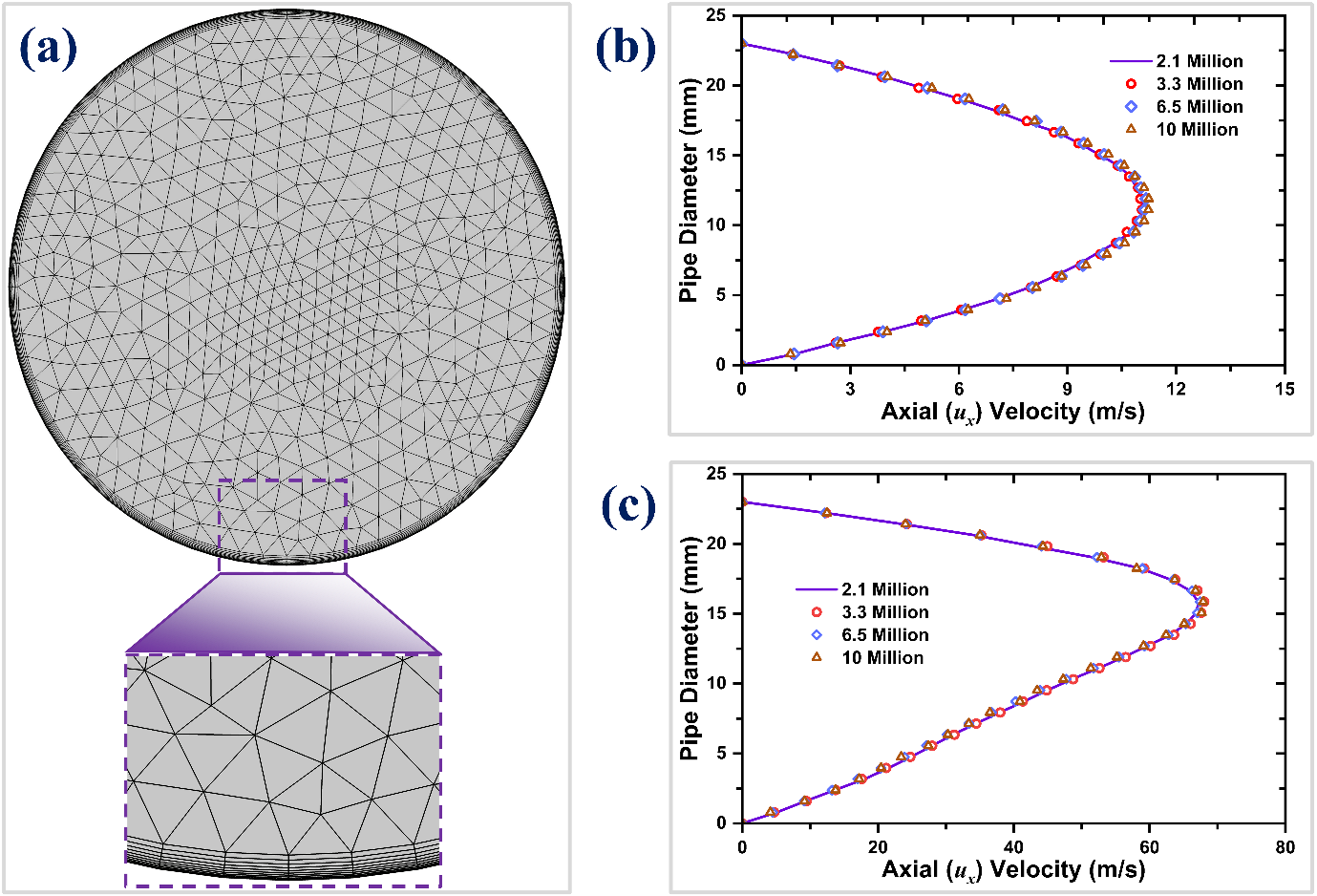


**Fig. S1.** **(a)** Computational meshed model, and Mesh independence test **(b)** Axial velocity (**𝑢*_x_***) profile at the onset of the heating zone, and **(c)** Axial velocity (**𝑢*_x_***) distribution evaluated at ½ D downstream of the substrate, illustrating convergence across mesh densities and confirming numerical stability under transient flow conditions.

**Section: S2 Transport vs reaction rate analysis**

Diffusion Coefficient (D) at 1000 K = 10^-4^ m^2^/s

Decomposition of Hexane at ~1000-1300 K = 0.05-0.02 m/s

Hexane is the heavier hydrocarbon, so its decomposition is generally faster than that of methane because:

1. C–C bonds break more easily
2. Pyrolysis occurs at lower activation energies.

Boundary Layer thickness L = 0.001 m

Damköhler number $Da=\frac{k_{r}L}{D}$

Da ≈ 0.2-0.5.

(Da ≈ 0.1-1 refers to mixed/transport-sensitive regime)

Da < 1 indicates that surface reaction rates are slower than mass transport rates; therefore, variations in near-wall transport can still significantly influence precursor supply and growth uniformity.

**Section: S3 Velocity field evolution with substrate inclination**

**Fig. S2 (a-d)** compares cross-sectional velocity fields evaluated on a plane located 10 mm downstream of the substrate center for tilt angles of 9°, 21°, 33°, and 45°, respectively. The downstream plane is decomposed into (i) velocity magnitude (**𝑢**), (ii) axial velocity (**𝑢*_x_***), (iii) vertical velocity (**𝑢*_y_***), and (iv) spanwise velocity (**𝑢*_z_***), enabling direct interrogation of wake deficits, recirculation signatures, and secondary-flow structure arising from substrate-flow interaction. For 9° tilt (**Fig. S2a**), the combined velocity field exhibits a comparatively weak velocity deficit, confined near the geometric center of the cross-section, with gradual lateral gradients and limited upward extension toward the tube wall. The absence of negative values in (**𝑢*_x_***) indicates that flow reversal is negligible at this downstream station. The (**𝑢*_y_***) contours display a low-intensity vertical dipole, with weak downward motion near the lower half and mild upward motion beneath the wake core, suggesting modest cross-stream redistribution [9]. Correspondingly, the (**𝑢*_z_***) field exhibits a diffuse and weakly organized counter-rotating vortex pair, indicating limited streamwise vorticity and subdued three-dimensionality of the wake [10,11]. At 21° tilt (**Fig. S2b**), the wake footprint in both (**𝑢**) and (**𝑢*_x_***) becomes more distinct and shifts upward toward the upper arc of the tube, reflecting stronger momentum extraction associated with increased streamline curvature at the inclined surface. Although reverse flow is not prominent, the axial velocity gradients are steeper around the wake boundary. The (**𝑢*_y_***) field reveals a clearer vertical dipole with enhanced magnitude, indicating stronger upwash beneath the wake and compensating downwash near the lower wall. In the (**𝑢*_z_***) component, the counter-rotating vortex pair becomes more coherent and laterally separated, consistent with the strengthening of streamwise vortical structures generated at the substrate-wall junction. For the 33° case (**Fig. S2c**), the axial-velocity deficit region remains well defined but is more laterally distributed, producing a broader low-velocity zone across the upper-central cross-section rather than a sharply localized core. The (**𝑢*_x_***) field shows smooth recovery without pronounced reverse-flow pockets at this station. The (**𝑢*_y_***) contours maintain a strong dipole structure, while the (**𝑢*_z_***) field shows expanded vortex lobes with reduced peak intensity compared with 45°, indicating that secondary vortices persist but are less concentrated and more spatially spread across the cross-section. In contrast, the 45° configuration (**Fig. S2d**) exhibits the most severe wake signature. The (**𝑢**) and (**𝑢*_x_***) maps show a deep and compact velocity deficit directly beneath the upper arc, accompanied by steep surrounding gradients and near-stagnation within the wake core. Localized regions of strongly reduced axial velocity indicate sustained separation-induced momentum loss under an adverse pressure gradient (APG), a canonical feature of separated bluff-body wakes in confined channels [12]. The (**𝑢*_y_***) component displays the strongest vertical dipole among all cases, signifying intensified downward and upward cross-stream motions linked to shear-layer roll-up. The (**𝑢*_z_***) contours reveal a compact, high-contrast counter-rotating vortex pair, characteristic of strengthened streamwise vortices persisting into the downstream wake.


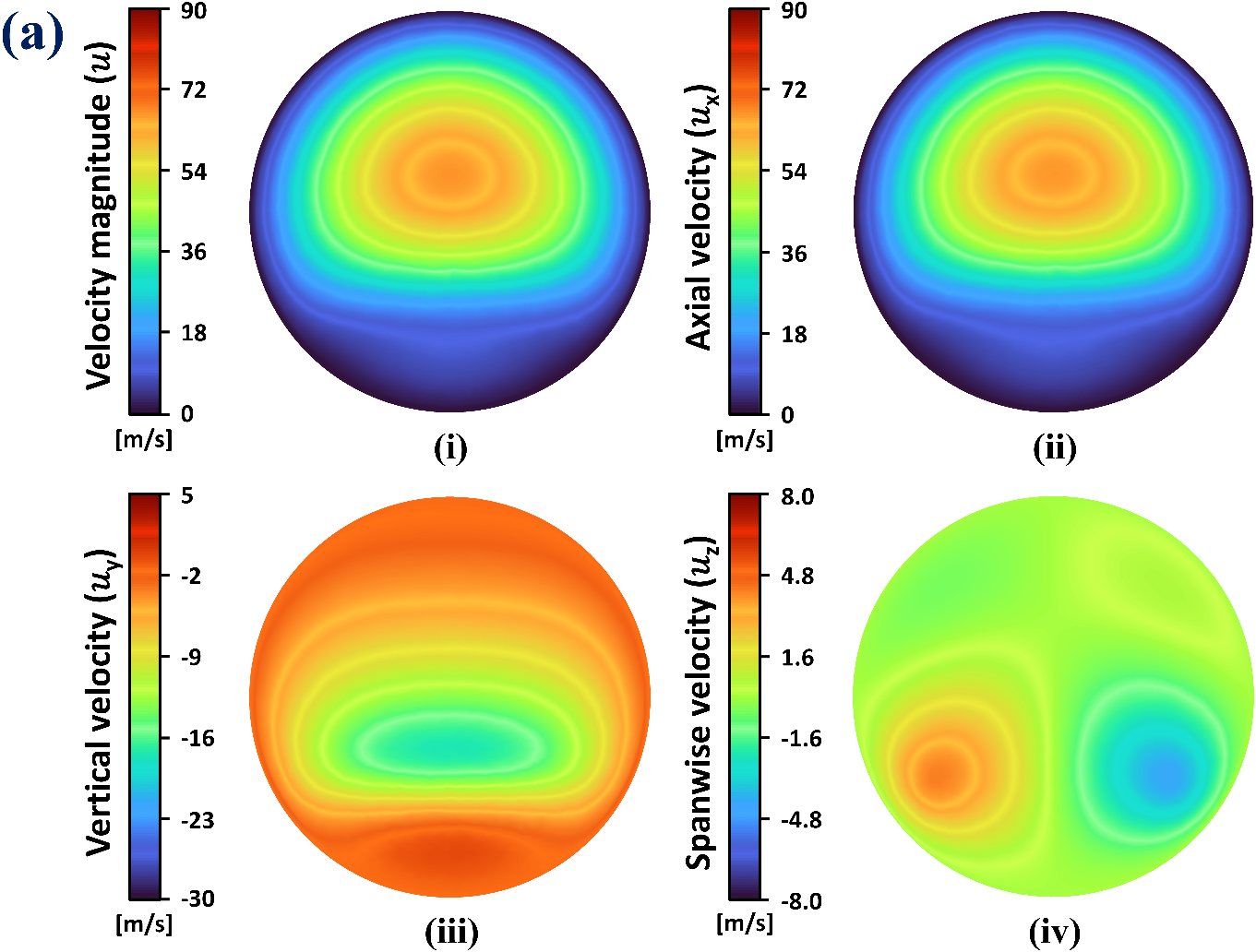


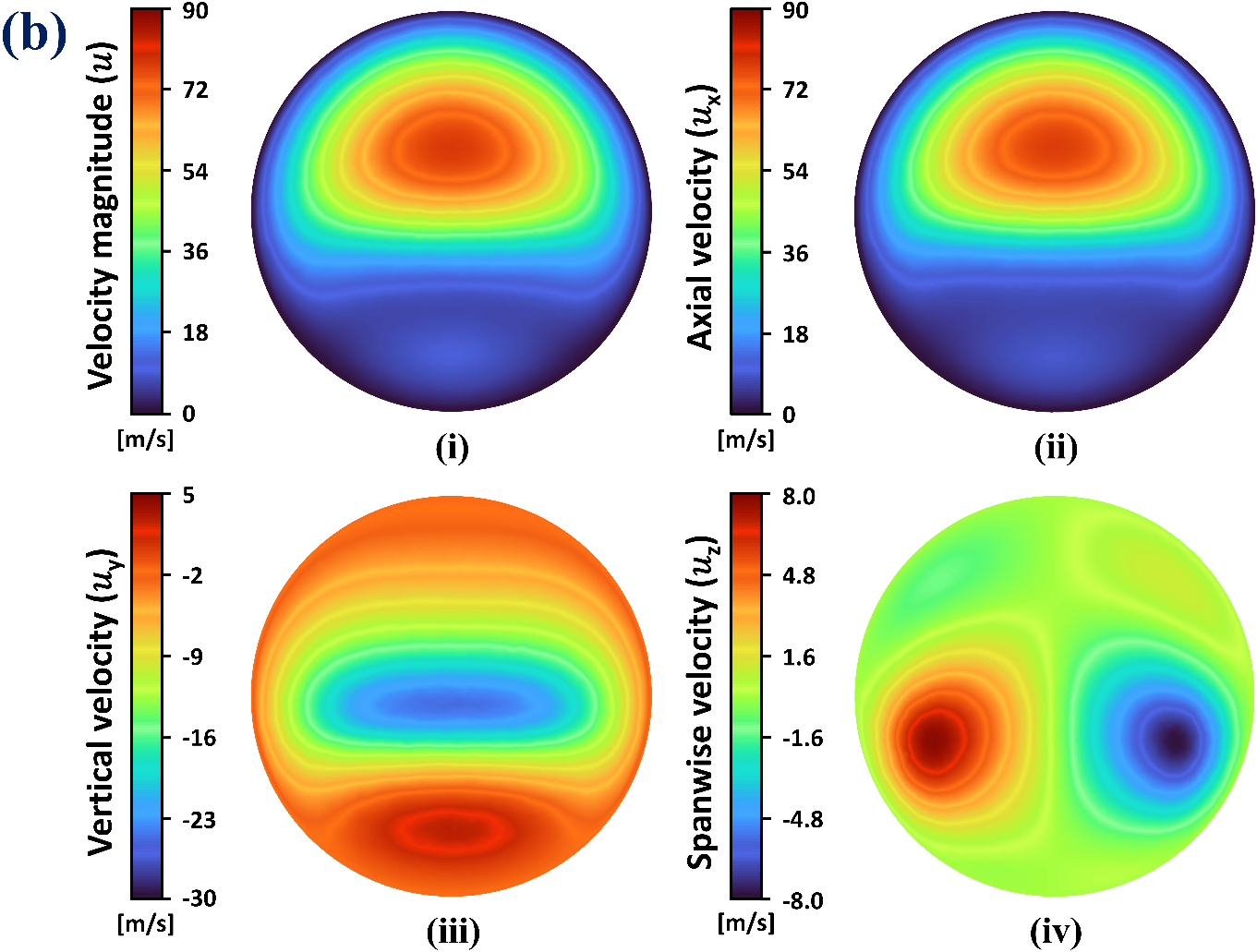


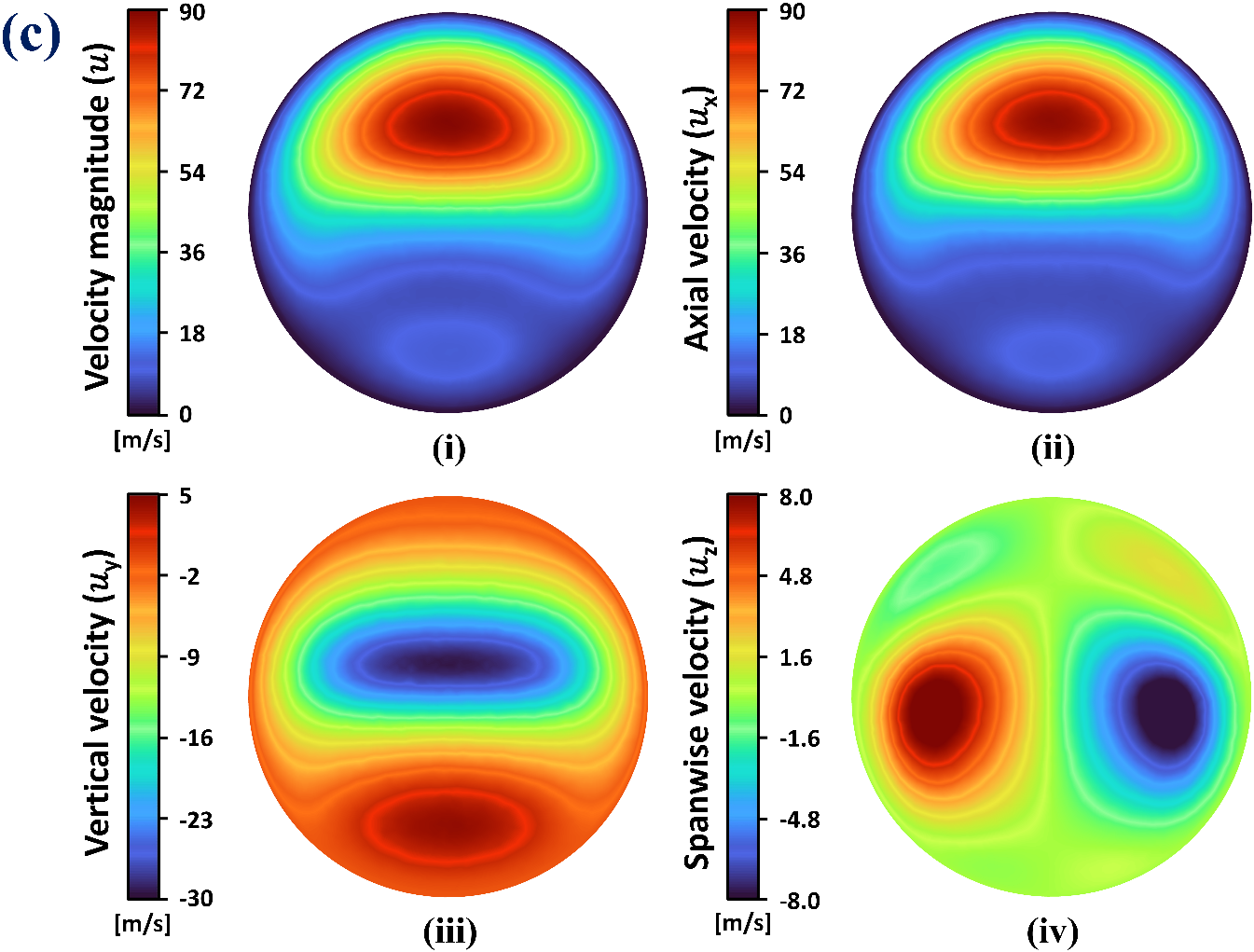


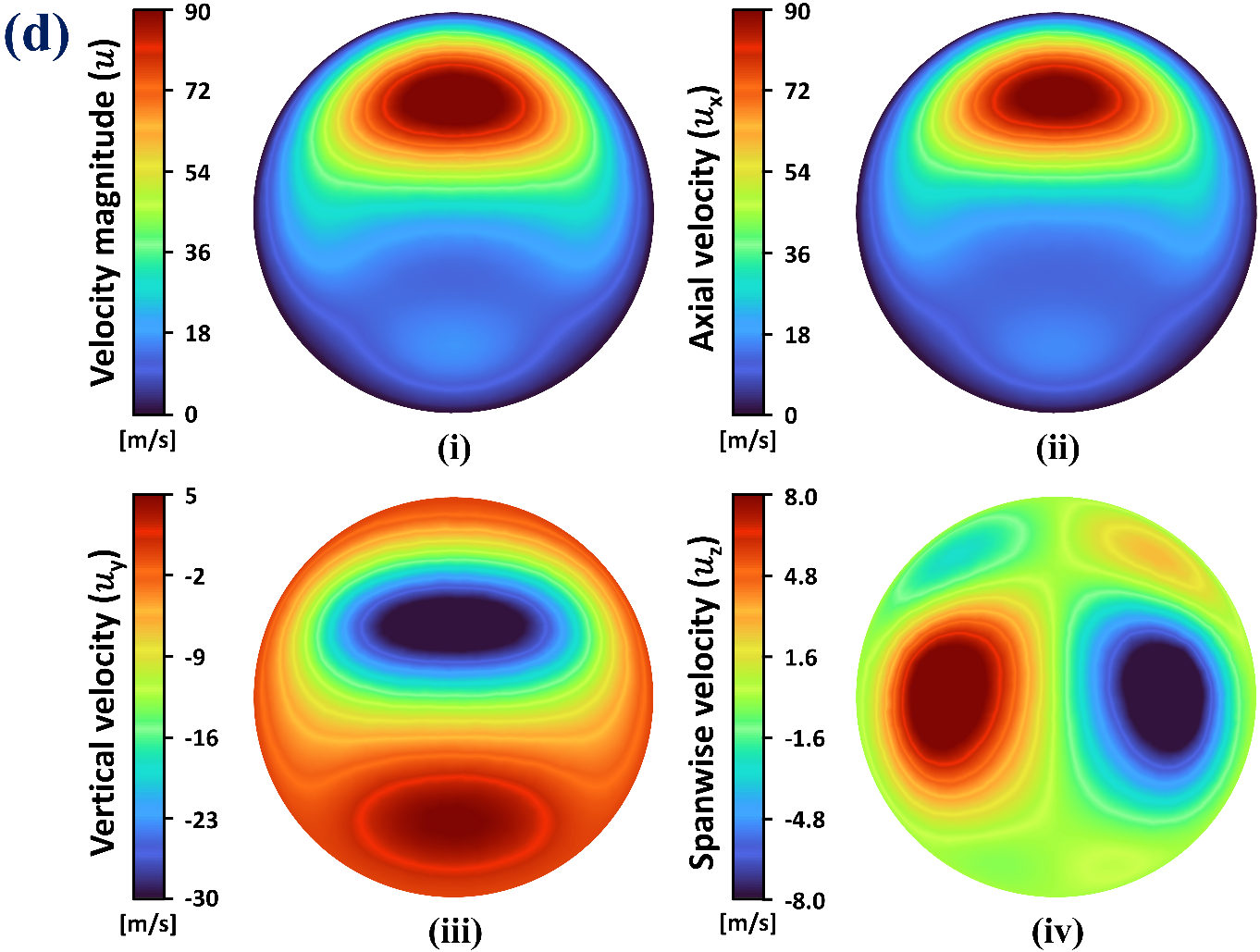


**Fig. S2.** Cross-sectional velocity fields evaluated at a plane located 10 mm downstream of the substrate center for substrate tilt angles of **(a)** 9°, **(b)** 21°, **(c)** 33°, and **(d)** 45° orientations. For each orientation, contours of (i) Velocity magnitude (**𝑢**), (ii) Axial-velocity (**𝑢*_x_***), (iii) Vertical-velocity (**𝑢*_y_***), and (iv) Spanwise-velocity (**𝑢*_z_***) are shown to illustrate downstream wake structure and secondary-flow development.

**References**

[1] J.H. Ferziger, M. Perić, R.L. Street, Computational Methods for Fluid Dynamics, 4th ed. 2020, Springer, Cham, 2020. https://doi.org/10.1007/978-3-319-99693-6.

[2] R.I. Issa, Solution of the implicitly discretised fluid flow equations by operator-splitting, Journal of Computational Physics 62 (1986) 40–65. https://doi.org/10.1016/0021-9991(86)90099-9.

[3] D. Costero, F. Piscaglia, Second-Order Time-Accurate ALE Schemes for Flow Computations with Moving and Topologically Changing Grids, Fluids 8 (2023) 177. https://doi.org/10.3390/fluids8060177.

[4] T. Joshi, O. Parkash, R.K.B. Gallegos, G. Krishan, Comparative investigation of the energy consumption and heat transfer characteristics of Uni-modal and Bi-modal slurry flow through a straight pipe, Ocean Engineering 316 (2025) 119987. https://doi.org/10.1016/j.oceaneng.2024.119987.

[5] T. Joshi, O. Parkash, R.K.B. Gallegos, G. Krishan, Computational investigation of transportation and thermal characteristics in a *bi*-modal slurry flow through a horizontally placed pipe bend, Powder Technology 442 (2024) 119879. https://doi.org/10.1016/j.powtec.2024.119879.

[6] T. Joshi, O. Parkash, R.K.B. Gallegos, G. Krishan, Parametric investigation of slurry transport: Computational insight into the impact of particle composition and Prandtl numbers, Physics of Fluids 36 (2024) 023308. https://doi.org/10.1063/5.0187126.

[7] T. Joshi, O. Parkash, A.A. Murthy, G. Krishan, Numerical investigation of Bi-model slurry transportation in a straight pipe, Results in Engineering 17 (2023) 100858. https://doi.org/10.1016/j.rineng.2022.100858.

[8] T. Joshi, O. Parkash, G. Krishan, A.A. Murthy, Numerical investigation of *Bi*-model slurry transportation through horizontal pipe bend, Powder Technology 418 (2023) 118284. https://doi.org/10.1016/j.powtec.2023.118284.

[9] S. Sen, S. Mittal, G. Biswas, Steady separated flow past a circular cylinder at low Reynolds numbers, Journal of Fluid Mechanics 620 (2009) 89–119. https://doi.org/10.1017/S0022112008004904.

[10] C. Polat, D.B. Saydam, M. Soyler, C. Ozalp, Horseshoe vortex formation around a rotationally oscillating cylinder: Experimental investigation with PIV, Ocean Engineering 336 (2025) 121666. https://doi.org/10.1016/j.oceaneng.2025.121666.

[11] Q. Liu, Wall-mounted circular cylinder flows, Physics of Fluids 36 (2024) 113620. https://doi.org/10.1063/5.0237808.

[12] D. Borgmann, S. Hosseinverdi, J. Little, H. Fasel, Experimental and numerical investigations of transition in a pressure-gradient-induced laminar separation bubble, Journal of Fluid Mechanics 1007 (2025) A23. https://doi.org/10.1017/jfm.2025.43.
